# Design of a Multipurpose Photonic Chip Architecture for THz Dual-Comb Spectrometers

**DOI:** 10.3390/s20216089

**Published:** 2020-10-27

**Authors:** Andrés Betancur-Pérez, Pedro Martín-Mateos, Cristina de Dios, Pablo Acedo

**Affiliations:** 1 Department of Electronics and Telecommunications, Instituto Tecnológico Metropolitano, 050013 Medellín, Colombia; andresbetancur@itm.edu.co; 2 Department of Electronic Technology, Universidad Carlos III de Madrid, 28915 Leganés, Spain; pmmateos@ing.uc3m.es (P.M.-M.); pag@ing.uc3m.es (P.A.)

**Keywords:** Dual-Comb, frequency shifter, optical combs, optical injection locking, photonic integrated circuit, THz spectroscopy

## Abstract

In this work, we present a multipurpose photonic integrated circuit capable of generating multiheterodyne complex Dual-Combs (DC) THz signals. Our work focuses on translating the functionality of an electro-optic tunable DC system into a photonic chip employing standard building blocks to ensure the scalability and cost efficiency of the integrated device. The architecture we analyze for integration is based on three stages: a seed comb, a mode selection stage and a DC stage. This final DC stage includes a frequency shifter, a key element to improve the final detection of the THz signals and obtain real-time operation. This investigation covers three key aspects: (1) a solution for comb line selection on GHz spaced combs using OIL or OPLL on photonic chips is studied and evaluated, (2) a simple and versatile scheme to produce a frequency shift using the double sideband suppressed carrier modulation technique and an asymmetric Mach Zehnder Interferometer to filter one of the sidebands is proposed, and (3) a multipurpose architecture that can offer a versatile effective device, moving from application-specific PICs to general-purpose PICs. Using the building blocks (BBs) available from an InP-based foundry, we obtained simulations that offer a high-quality Dual-Comb frequency shifted signal with a side mode suppression ratio around 21 dB, and 41 dB after photodetection with an intermediate frequency of 1 MHz. We tested our system to generate a Dual-Comb with 10 kHz of frequency spacing and an OOK modulation with 5 Gbps which can be down-converted to the THz range by a square law detector. It is also important to note that the presented architecture is multipurpose and can also be applied to THz communications. This design is a step to enable a commercial THz photonic chip for multiple applications such as THz spectroscopy, THz multispectral imaging and THz telecommunications and offers the possibility of being fabricated in a multi-project wafer.

## 1. Introduction

Current THz technology has given birth to a new wave of applications in important fields such as medicine, security, telecommunications and industry, but, specifically, THz radiation has gained special importance for science and the industrial sector to recognize and characterize non-polar materials, plastics and gases [1,2,3,4,5,6,7,8]. To achieve this, it mainly required a spectrometer capable of interrogating samples to extract information like the real and imaginary parts of the refractive index [9]. In this respect, we now have commercially available frequency sweep-based THz spectroscopy devices at our disposal, however, they are not suited for applications requiring real time measurement. To enable such real time processing, we need to interrogate a huge spectral range at once and a high-speed digital signal processor (DSP) to deal with a broadband signal, and this means an expensive solution. To solve this limitation, in 2002 Schiller proposed a novel approach for infrared spectroscopy, which makes use of two frequency combs (now known as Dual-Comb) [10], with a kHz difference in their repetition frequency $\Delta f_{r}$. These combs interrogate a wide spectral range in the sample under test. Then, the power of each of the comb teeth is absorbed according to their frequency, shaping the envelope of the spectrum and describing the absorption fingerprint of the studied component, in a discretized manner. When the two combs beat each other in a square law detector (i.e., photomixing devices), even if the repetition frequencies $f_{r1}$ and $f_{r2}$ have values in the order of GHz, the result is a new comb with comb lines spaced $\Delta f_{r}$ (in the order of kHz), centered in a desired intermediate frequency $f_{IF}$. Some upper frequency combs created are naturally filtered by the frequency response of the detector. This signal is no longer broadband; thus, the main advantage is the acquisition of a shrunken comb, and then it can be sampled and computed with cheaper, commercial, off-the-shelf digital signal processors.

In ref. [11,12,13], Dual Combs were implemented in the THz domain using quantum Cascade Lasers (QCL). They offer interesting features like (a) broad spam combs, (b) high Quantum efficiency, (c) narrow linewidth emissions, (d) simplicity and compactness. However, they are difficult to control and reconfigure, given their need for a sub-zero temperature to work properly. However, this approach is constantly improving, as the work proposed in ref. [14] shows. In ref. [15], an interesting THz Time-Domain (TDS) spectrometer referred to as Asynchronous Optical Sampling (ASOPS) was implemented. This architecture can tune its resolution down to sub-MHz. The resolution of TDS systems depends on the size of the window used (number of pulses acquired) to obtain narrow lines in the spectrum after numerically applying Fourier transform. As a result, the acquisition time is extended according to the time needed to reach a certain resolution. On the other hand, TDS systems are benchtop implementations which makes them difficult to distribute widely in the world as a commercial product. In [16], we implemented a THz Dual-Comb Spectrometer (TDCS) using Optical Injection Locking (OIL) and electro-optical devices. This setup offers several advantages: (a) tunable resolution, (b) tunable frequency range, (c) easy operation and (d) enable real-time interrogation. Nevertheless, there are some limitations like the sensitivity to the difference in the waveguide paths, since the architecture is using Mach Zehnder interferometers (MZI), and related to this issue is the difference in the temperature of each of the bulky components and optical fibers present on the system. This scheme depends on the comb spam generated with an electro-optical phase modulator or several of them. The TDCS architecture was tested on the mm-wave spectral range using a 100-GHz photodiode on the transmitting side, and a Schottky diode on the detection side. We could follow the frequency response of the device under test (DUT), and these results reproduced the specs provided by the manufacturer. This technique was also implemented in [17,18], covering the range of 0.1–0.6 THz by using two cascaded phase modulators. In [19] we measure the absorption line of ammonia for as fast as 1 ms of interrogation time, and the bandwidth interrogated depends on the comb span generated with the DC generator. With this in mind, we think there are potential applications where fast interrogation and a high resolution is a priority. For instance, real-time tracking of water emulsions in the oil industry, gas monitoring or moisture content on plastic components.

In this work, we propose a version of the TDCS presented in ref. [16,17,18,19], integrated in an InP Photonic Integrated Circuit (PIC). Moreover, the same PIC can be used as a THz or optical communication transmitter. To take such a system into a chip we must face some challenges: (1) enabling comb line selection over an optical comb signal with GHz spacing, (2) enabling frequency shifting in a PIC, and (3) building the whole system with a minimum of electrodes. We intend to transfer all the benefits of this flexible scheme into a chip, but with all the limitations mitigated. Our starting point is a fully tested benchtop scheme. By miniaturizing it in a PIC, we could go a step further to fulfill a compact and commercial THz Dual-Comb System for multiple purposes such as, spectroscopy, THz multispectral imaging and telecommunications. Our architecture is, to the best of our knowledge, a novel architecture to be integrated in a PIC. Given that there is no Building Block (BB) in a foundry capable of frequency shifting, we propose a unit cell capable of creating the same effect using the process design kits (PDKs) provided by an InP-based foundry, different to the Single Side Band Suppressed Carrier (SSB-SC) approach suggested in ref. [20], which is a more complex system and difficult to operate. By using official PDKs, we can easily pass our design to a multi-project wafer (MPW) for fabrication. This paper will be structured as follows. In the first part, we will provide the overall architecture of the TDCS with its working principle, and it will be placed in contrast with the architecture we provide for photonic integration. Next, we will describe the system stage by stage. In the third part, we will prove the functionality of the frequency shifter we suggest for the TDCS. In the last part we will discuss and present our conclusions.

## 2. TDCS Architecture

**Figure 1:**
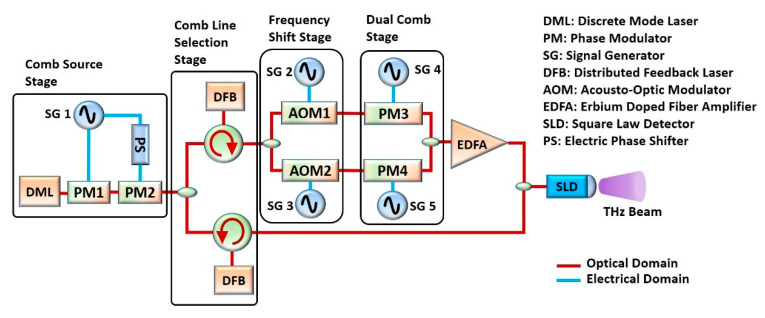

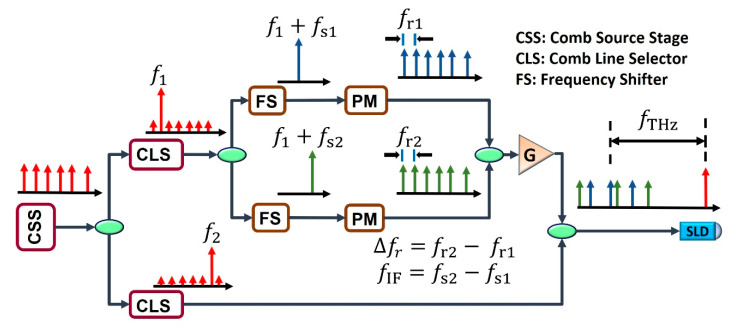
(**Up**) System Architecture of a benchtop THz Dual-Comb Spectrometer (TDCS). In the “comb source stage” we generate an optical comb using a DML and two cascaded phase modulators (PMs) operated with a signal generator. The PS is used to widen the optical comb produced for the first PM. The output of this stage is divided for an optical coupler and both signals enter the “comb line selection stage” where two optical circulators are used with distributed feedback (DFB) lasers to produce the OIL phenomenon. The output of the upper branch of the stage passes through the “frequency shift stage” where AOMs are used to shift the incoming signal frequency. Next, the signal passes through the “dual comb stage” where two phase modulators produce two optical combs with $\Delta f_{r}$ of difference in their repetition frequency. Given the several stages the upper signal needs to get through, an EDFA is placed to compensate insertion losses. At the end, the signal is combined with the locked signal coming from the lower branch of the “comb line selection stage” and they are mixed in a square law detector (SLD) to downconvert the signal in the THz range. (**Down**) Signal behavior.

**Figure 2:**
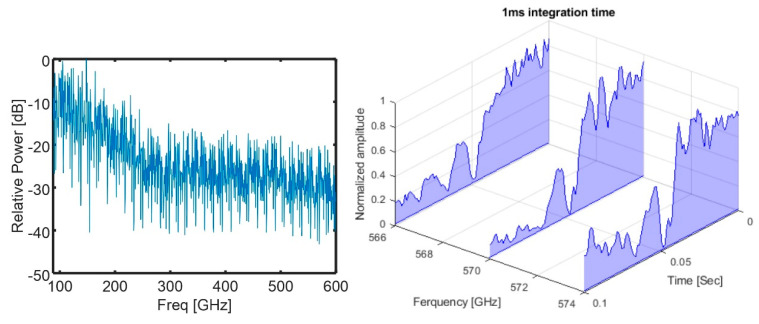
**Left**, sampled spectrum from 100 to 600 GHz. **Right**, three comb lines used to track, in real time, the Ammonia absorption line around 570 GHz.

In Figure 1, the benchtop TDCS that serves as a reference for the integrated architecture presented in this work is depicted [16]. In this system, a Narrow Linewidth Discrete Mode Laser is used as the optical source to create an initial seed optical comb using a cascade of electro-optic phase modulators. This stage is named in Figure 1 as “comb source stage”. The optical injection locking technique is then applied using slave Distributed Feedback Lasers (DFBs) to select and amplify two coherent lines from the initial seed comb. This is the second stage, depicted in Figure 1 as the “comb line selection stage”. At the output of this stage, the selected modes exhibited a Side-Mode Suppression Ratio (SMSR) of 27 dB. Next, one of the branches of the scheme takes the incoming beam and makes it pass through a stage called thte “frequency shift stage” (see Figure 1) to generate a proper intermediate frequency (IF). On this stage, an acousto-optic modulator (AOM) was used; however, if lower IF frequency is needed, two AOMs can be used, and they can be modulated with a difference in frequency equal to the desired IF, which must be in the range of the electrical frequency response of the THz detector. After the previous stage, the beam enters the “Dual-Comb stage”, where two phase modulators are operated with two RF tones with a difference in frequency, $\Delta f_{r}$, which is the repetition frequency of the comb in the IF. At the end of the scheme, a square law detector (SLD) is used to generate the THz signal by means of difference frequency generation [21], and at the receiving side, we use a THz detector whose specifications will vary according to the applications. For instance, if sensitive detectors are required, there are sensors with a low video-bandwidth but with higher responsivity. If bandwidth is needed, sensitivity will be low. This is due to a compromise between the video-bandwidth and sensitivity in the THz detectors [22,23,24]. Then, one kind of detector is used for instrumentation (to improve dynamic range) and the others are suitable for telecommunications (since, in this kind of scenario, systems need to deal with broadband signals) [25,26,27]. In companies like Toptica or Virginia Diodes, we can find two versions of the same Schottky receiver, one with higher bandwidth, higher noise equivalent power and lower responsivity. The other version offers the opposite parameters. In ref. [19], we used a photomixer to combine and send the two combs through the sample under test and a Schottky diode detector at the receiving side. In [18], we implemented a pair of photoconductive antennas using one of the combs as a local oscillator on the receiver. The results are summarized in Figure 2.

**Figure 3:**
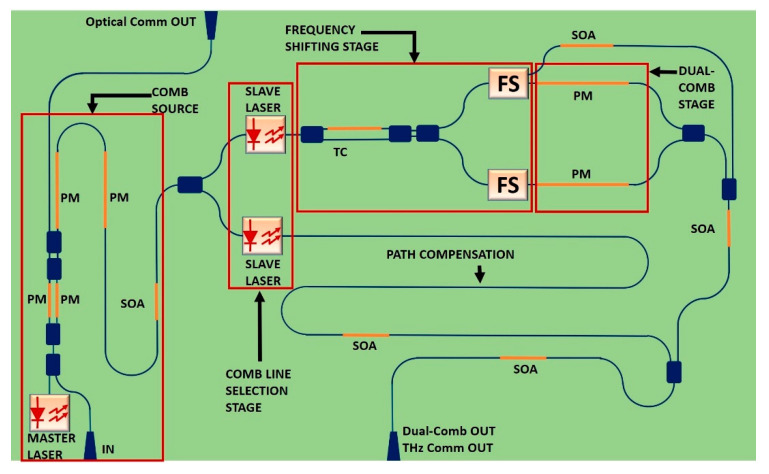
Integrated TDCS. PM: Phase modulator. SOA: Semiconductor optical amplifier. TC: Tunable coupler. PM: Phase modulator. FS: Frequency shifter. The operation is analogous to the benchtop system. In the comb source stage, the integrated laser can be selected as the master laser or a discrete laser beam injected in the “In” port. The input beam can be modulated by a Mach Zehnder structure composed of two parallel PMs, for optical communications. On the chip, several SOAs are used to compensate the losses of the chip waveguides. In the “comb line selection stage” there are no circulators, but photonic integrated lasers can lock the incoming beams by themselves. In the FS, a TC is added to select the operation mode of the chip (Dual-Comb mode or telecommunications mode). The upper FS provides two outputs to differentiate the Dual-Comb signal from the telecommunications signal. The “path compensation” waveguide is for balancing the optical paths of the Mach Zehnder structure.

With the aim of describing the whole system in a simpler manner, we will divide it by stages, as named in Figure 1. The whole architecture for the PIC version of the TDCS is illustrated in Figure 3. This system is a double-purpose photonic integrated chip, capable of generating THz Dual-Combs for spectroscopy and OOK signals for telecommunications, where the Tunable Coupler (TC) has the role of switching the incoming light at will to select the mode of operation as a TDCS or as a THz transmitter. The components used for the TC are a 1 × 2 MMI coupler, a 2 × 2 MMI coupler, a 1mm-long phase shifter and a 1-mm-long waveguide. By applying a voltage on the phase shifter, we can control the phase of the respective branch and, in this way, we can adjust the transmitted power on the two output ports by inducing a constructive interference in one port and a destructive interference on the second port. If the coupling ratio of the TC is configured to be 0.5, then we can obtain a Dual-Comb signal. On the other hand, if we configure on the TC a coupling ratio equal to 1, then all the power will go through one of the frequency shifters (FS). The FS is based on the double side band suppressed carrier modulation (DSB-SC); therefore, we can bias the associated phase modulator to produce an On–Off Keying (OOK) modulation, or even PAM-4 or higher-order modulation formats. A Semiconductor Optical Amplifier (SOA) is placed after the DSB-SC stage to amplify the signal in the case of Dual-Comb operation, or we can bias the SOA to absorb the incoming power and let the power remaining follow the path related to THz communications operation. The details of the FS are examined in a further section.

### 2.1. Comb Source Stage

In ref. [28], a comb generator was integrated with InP technology. In this work, the comb generator was implemented by cascading Phase modulators, and as an optical source, a Distributed Bragg Reflector (DBR) laser was integrated with an alternative to inject light from an external discrete laser. It was reported that this monolithic tunable optical comb generator created, at its output, 28 comb lines using a 5 dB criteria and repetition frequencies around 5 GHz. It should be noted that the modulation depth of the implemented phase modulators can be improved if capacitive-loaded traveling-wave electrodes [29,30,31] are used, and, according to the tests run, more voltage can be applied, hence a wider comb-span can be achieved. The main function of this stage is to generate a comb as wide as possible, to reach a span in the order of THz, as made in ref. [18].

### 2.2. Comb Line Selection Stage

**Figure 4:**
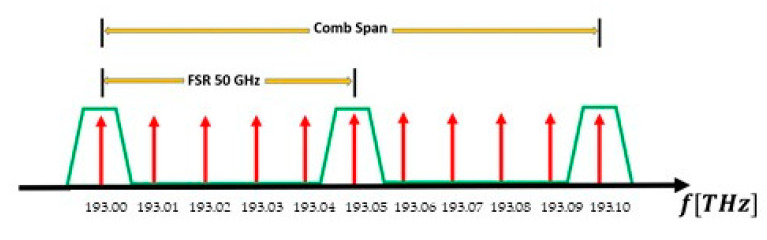
Illustration of the filter designed when the Comb Span is greater than the FSR of the filter. We look for just one comb line, and more than one is selected. This is an example of a comb with 10 GHz frequency spacing.

There are several ways to filter a signal to acquire the range of interest. In this case, we discuss optical combs with comb line spacing in the order of GHz. If we try optical band pass filters, we must ensure a narrow band characteristic that fits the frequency spacing of the optical comb and a wide band stop frequency range. In this way, selecting more than two comb lines is avoided, as shown in Figure 4. Thus, a condition to be satisfied by the filters is that the free spectral range (FSR) must be greater than the OFC span. However, in order to reach such behavior, a high-order optical filter should be implemented, and the most sophisticated structure is the ARMA filter [32,33]. An ARMA filter can reach a narrow bandpass frequency response, but its nonlinear phase behavior [34,35] is not reliable for THz spectroscopy, where the phase is important to maintain the coherence when beating two optical beams in an SLD [36]. Then, using an optical ARMA filter is not worth considering for photonic-integrated TDCS. For instance, a filter with a bandwidth near 5 GHz, and 10% of the FSR will select another comb line of 50 GHz [37]. Another fact of the photonic integrated filters is the influence of the losses in the coefficients of the transfer function. This characteristic is more intense when using InP technology [38]. In order to prevent this, semiconductor optical amplifiers (SOA) are placed strategically so the losses are compensated [39]. This issue is also linked to the FSR and the extinction ratio. If a lower FSR is required, this implies longer paths with bigger losses in one of the branches of the Asymmetric MZI (AMZI) interferometer, and therefore the extinction ratio between the bandpass and band stop regions will be lower.

**Figure 5:**
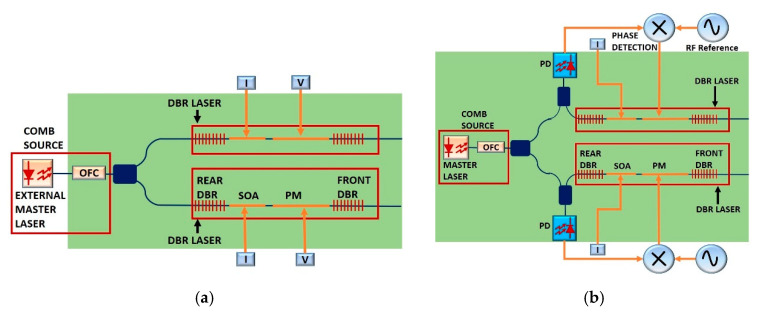
(**a**) Scheme of a photonic integrated version of the optical injection locking technique, (**b**) Scheme of an Optical Phase Locked Loop. The slave DBR lasers are used to lock one of the comb lines of the Optical Frequency Comb (OFC). The integrated DBR lasers can tune the wavelength by varying the current I on the SOA and the voltage V on the PM. The PDs on the OPLL are used to mix the master and slave beams to detect in the electrical domain the phase difference. The rear and front DBR are made of WBGs.

One aspect to consider in this system is the complexity. There are other solutions to selecting one single line of the comb. One of those solutions is using integrated waveguide Bragg gratings (WBG); however, this implies the use of experimental structures capable of tuning the central wavelength, and to date, there is no building block available on the foundries [40,41,42,43,44] which can be implemented on an MPW, even though they are key components in building high-quality on-chip lasers [28,45]. We can build tunable photonic integrated lasers using WBGs and, with these, we can use the OIL technique or enable optical phase locked loops (OPLLs). There are several advantages of using slave lasers as a mean to select comb lines: (a) we can lock a desired comb line, rejecting the others with a high SMSR, (b) slave lasers amplify the desired comb line, (c) they have wide tunability, and (d) this ensures coherence between the two comb lines selected for an optimized beating on the SLD [46]. We suggest two architectures using slave lasers and they are depicted in Figure 5.

In Figure 5a, the OIL scheme that was proved in ref. [47] can be observed, and the respective slave lasers are represented by two DBR lasers instead. Using OIL on a PIC suggests the use of an external optical source with an isolator integrated on it. Given that the integrated lasers emit radiation in both directions, the beam propagating backwards can induce noise on an integrated master laser, therefore, implementing a discrete laser component yields the benefit of including an isolator. This modification is a consequence of not counting BBs for optical isolators; nevertheless, there is some research aiming to enable such functionality [48]. An OPLL is illustrated in Figure 5b, which can be used to lock the comb line of interest. As can be seen, in this scheme we can include the master laser in the same chip because there is no direct path connecting it with the slave lasers; in this manner, we can skip the possibility of generating noise inside the master laser. In ref. [49], an OPLL was reported for comb line selection with an attenuation of noise and sidebands above 58 dB, with frequency offsets near 12 GHz. The OPLL offers wide tunability and the measured phase noise was below −100 dBc/Hz. The comb source stage can be designed as in ref. [28] to allow one external laser or an integrated one, if the schemes of Figure 5a,b are available. According with the measurements made in ref. [16], when a whole optical comb is injected in a slave laser, there are residual sidebands which were mitigated, in relation to the comb lines locked, by about 30 dB. The rejection of the undesired sidebands depends on the frequency spacing among the comb lines, the injection ratio used on the slave laser and the frequency detuning. If the laser is optimized to accept larger frequency detuning, then the attenuation of the sidebands will be reduced. If OIL is operated with a reduced frequency detuning tolerance, then the sidebands will be more attenuated. We are designing this PIC taking the complexity of the system as an important rule. In this regard, an OIL subsystem offers a simple operation and reduced complexity compared to an OPLL. On the other hand, we can reach a considerable SMSR for the generation of high-quality signals in the THz domain. With an OPLL, we obtain greater complexity because it requires an external control loop system where its loop delay limits the possible linewidth of the master laser and, therefore, an OPLL will demand expensive electrical systems for fast and stable operation.

### 2.3. Frequency Shifting and Dual-Comb Stage

One important functionality for optical signal processing is the frequency shifting. Hence, it is indispensable to count with this function in PICs if we seek to enable a wider range of applications. To date, there is no BB on any foundry capable of executing a frequency shift in an optical beam. Nonetheless, there are different investigations looking at such a goal [50,51,52,53]. One concept used for frequency shifting on PICs is the single sideband suppressed carrier modulation, where an IQ-MZM is used to create an amplitude modulation in the Mach Zehnder Modulators (MZM), and then SOAs and phase shifters are tuned so the sidebands cancel each other, and the tone of interest remains with greater amplitude compared to the others. These systems work properly by seeking the mitigation of several sidebands whose phase response is different when the phase shifters are modified. Therefore, the problem is scattered in the mitigation of certain amount of tones present on the process.

**Figure 6:**
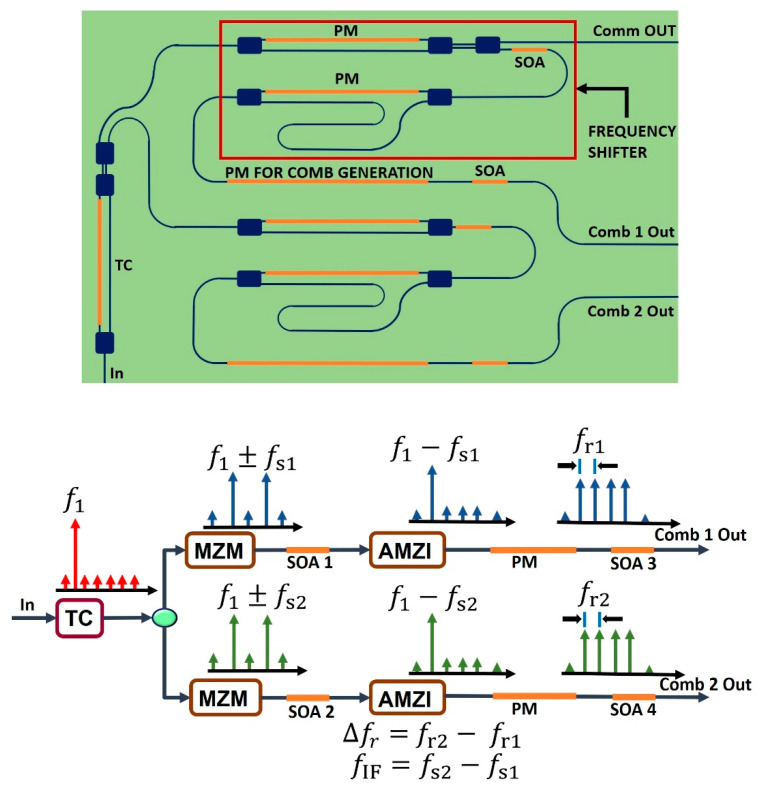
(**Up**) Architecture of the frequency shifter and Dual-Comb stages. The TC is used to select the operation mode. The SOA of the frequency shifter can be biased to absorb the power and prevent the doubling of the telecommunication signal. The first PM comprises the MZM. The second one comprises the AMZI. The lower branch is a copy of the upper branch. The two frequency shifters comprise the “frequency shift stage”. The “PM for comb generation” placed in the upper and lower branches comprise the dual comb stage. (**Down**) Signal Behavior.

We designed a simpler structure to shift the frequency based on a double side band suppressed carrier approach (DSB-SC). In this approach, we concentrated our effort on the cancelation of one single tone of the DSB-SC modulation. The scale of the frequency change is in the order of GHz, even though we induced a slight frequency difference between two paths to seek a desired IF frequency to which the video-bandwidth of available THz detector can respond. After the frequency shifter, a phase modulator can be found, with the purpose of generating an optical comb. The architecture of the PIC frequency shifter is depicted in Figure 6.

In the frequency shifter, a Mach Zehnder Interferometer can be seen, where a bias is applied to operations in the lowest transmission regime. In this operation point, we ensure a DSB-SC modulation. In the next stage, an Asymmetric Mach-Zehnder Interferometer (AMZI) is used with the aim of mitigating the amplitude of one of the sidebands. In the output of the frequency shifter, a phase shifter is found, which is used for the generation of an optical comb. Between the previous two stages and at the output of the system, the optical signal passes through SOAs to compensate the losses. Moreover, the SOAs can be reverse-biased to absorb the remaining power of the two FS and, in this manner, prevent a hybrid mode of operation (i.e., Dual-Comb and THz transmitter at the same time). In the frequency shifter system, we focus on obtaining a design with a low complexity, considering the number of electrodes needed to produce the desired effect.

## 3. Simulation Results

To test our design, we used the Optsim simulation platform of Synopsys and the process design kits (PDKs) from an InP technology-based Foundry. All the phase shifters in the architecture were designed with a length of 2 mm to ensure a 180° phase with a voltage Vπ inside the range of the foundry recommendations. On the other hand, the phase shifter in the AMZI has the purpose of producing a greater tunability so that we can minimize one of the sidebands of the DSB-SC modulation. In order to make the filtering of the sideband by the AMZI easier, the modulation frequency used was 5 GHz and the FSR of the AMZI was 32.8 GHz, which means a length difference near to 3 mm and considering a group index ng = 3.05, specified by the foundry. We used deep etched components on the circuit to prevent light coupling between the waveguides, permit a closer distance among them, and allow a lower bending radius on the waveguides.

**Figure 7:**
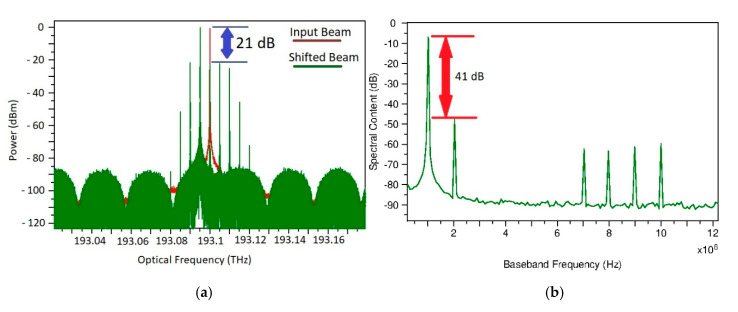
Normalized power spectra. (**a**) Resulting optical signal after a frequency shift. (**b**) IF signal after photodetection.

The length defined for all the SOAs was 200 µm with 60 mA of bias current. The voltage of the AMZI was tuned until one of the sidebands reached the minimum transmission. The Side-Band Suppression Ratio (SBSR) acquired was about 21 dB, as can be seen in Figure 7a. Using the scheme shown in Figure 5, and with a modulation frequency of 5.001 GHz on the lower structure, we could get an IF frequency of 1 MHz, which is inside the electrical frequency response of the available sensitive THz detectors. After the optical signal is processed, the spurious sidebands are created in the electrical domain higher-order harmonics (See Figure 7b), however, we expect that they will be naturally filtered if a 2 MHz video bandwidth THz detector is used. For our design, we prefer highly sensitive THz detectors so we can increase the dynamic range of the spectrometer.

**Figure 8:**
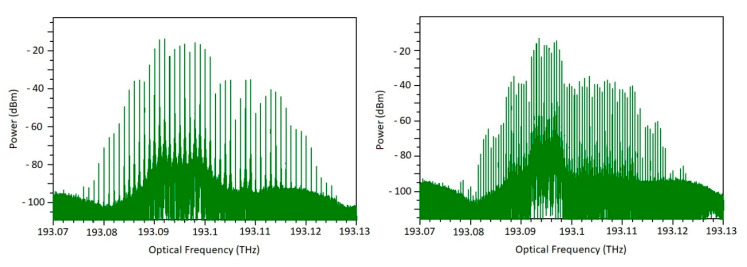
Optical comb after frequency shift. Resolution tuning capability. **Left**: 1 GHz Frequency spacing. **Right**: 500 MHz frequency spacing. Density of the of the comb is varied for increased resolution in the interrogation of a spectrometer.

**Figure 9:**
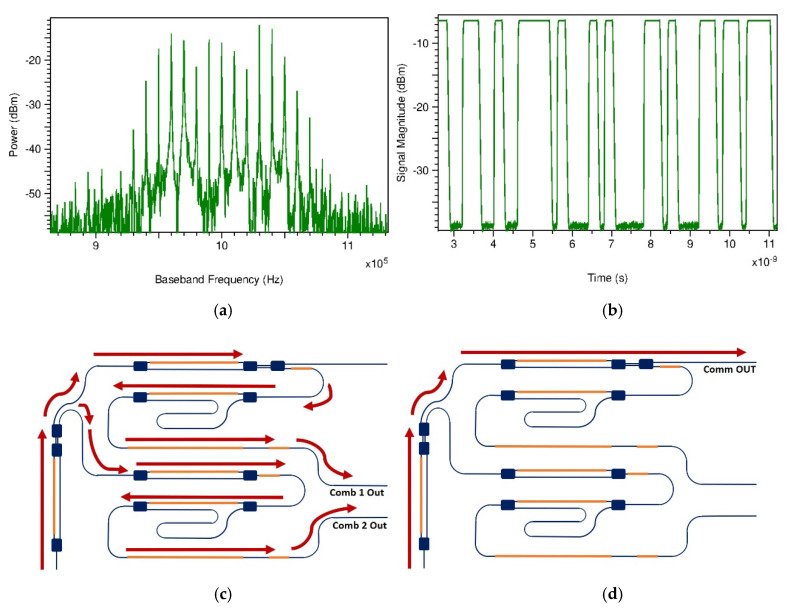
(**a**) Dual-Comb signal after photodetection. Comb centered on IF frequency. (**b**) Optical OOK signal when modulating MZM by PRBS. (**c**) Path followed by the light to obtain the Dual-Comb signal on (**a**). (**d**) Path followed by the light to obtain the OOK signal on (**b**).

In order to test the architecture of the frequency shifter when it is applied on the generation of a Dual-Comb, we use a modulation frequency equivalent to 1 GHz for one phase shifter and, for the other one, a frequency equivalent to 1 + 10 kHz. The IF frequency was centered on 1 MHz. The amplitude of the RF frequency was 14 volts on both PMs. The modulation depth can be increased if capacitive-loaded traveling-wave electrodes are used, and the length of the PMs are increased. We tuned the frequency spacing of the combs; thus, we can tune the resolution of a spectrometer. In Figure 8, the optical comb is depicted with 500 MHz and 1 GHz of repetition frequency. To test the designed FS, we used a PIN photodetector to beat the incoming beam and to generate the electrical signal in IF frequency. This signal is presented in Figure 9a. The photodetector was parametrized with a dark current of 1 µA. RIN, Shot, and spontaneous emission noises were enabled. Responsivity was modified to 0.8 A/W. We switched the TC to operate the FS as an optical modulator for telecommunications. Figure 9b shows a signal corresponding to an electro-optic amplitude modulation. For this, the MZM used on the first stage is operated with a Pseudo-Random Binary Sequence (PRBS) at a bit rate equal to 5 Gbps with an offset Voltage to ensure an adequate OOK operation (i.e., linear region of the MZM characteristic curve). The SOA 1 at the MZM output is reverse-biased with 150 mA to prevent simultaneous operation. This signal can be mixed in a SLD (like an Unitraveling-Carrier photodiode UTC-PD) with another optical beam to downconvert the signal into the THz range for THz or mm-wave communications.

## 4. Conclusions

A novel design of a photonic integrated THz Dual-Comb spectroscopy was presented. This system can also be used for THz communications by switching a tunable coupler and changing the operation point of the FS. We demonstrated the possibility of fully integrating this system in a PIC, given that we can produce, with essential BBs, a frequency shift, ensuring the possibility of fabricating the design in an MPW of an InP-based foundry. The operation point of the FS is easy to find and requires a reduced complexity compared to the SSB-SC approach. We presented a PIC architecture which can select a single comb line from an optical comb with GHz spacing, and produce a frequency shift with a reduced number of electrodes. As we could envision, the spurious sidebands of the technique used produce more than one comb centered on higher IF frequencies, but we can harness the low bandwidth characteristic of sensitive THz detectors to filter the higher-order combs. Considering that the equivalent noise power is proportional to the system bandwidth, we can improve the signal to noise ratio of the spectrometer by fitting the IF comb span obtained with the Dual-Comb technique within the video bandwidth of the THz detector. In ref. [54], a THz CMOS detector with an integrated slot antenna and impedance-transforming elements was used. Its respective optical sensitivity was above 55 kV/W and its optical NEP < 20.8 pW/Hz at a full 3-dB bandwidth of 42 GHz. This setup presented an averaged signal to noise ratio around 89 for an integration time of 200 ms when a sample with 0.15 transmittance is measured. On the other hand, we should avoid using a too narrow band electrical frequency response THz detector since we do not want filtered comb teeth. We can obtain Hz-spaced combs, but this will demand more computation given the resolution and very stable frequency synthesizers.

For the generation of the combs, we need to be cautious with the voltage applied over the phase shifters, because a larger modulation index means more comb lines, but the dynamic range will decrease in the spectroscope, however, we believe that using a lock-in amplifier in an experimental setup of the PIC will improve the performance of the signal when deploying measurements. In future activities, the frequency shifter design is planned to be sent for fabrication, and in this way, we can verify its functionality and the results obtained in this research work.
